# Oral Health & Cognitive Impairment: Biological, Clinical, and Social Perspectives

**DOI:** 10.1093/geroni/igaf122.1222

**Published:** 2025-12-31

**Authors:** Bei Wu, Zhang Zhang, Huabin Luo

## Abstract

This symposium offers an in-depth exploration of the complex interplay between oral health and cognitive function in older adults, integrating biological, clinical, and social perspectives to deliver observational and behavioral insights. The first presentation investigates the biological underpinnings, examining how oral bacteria moderate the link between tooth loss and cognitive performance. It reveals that a high periodontal bacterial load significantly worsens cognitive dysfunction, even with minimal tooth loss, emphasizing oral hygiene’s critical role. The second presentation provides clinical evidence, assessing how multiple oral health issues—such as tooth loss, dental plaque, and periodontal symptoms—contribute to domain-specific cognitive decline, notably impacting memory and executive function. The third presentation explores behavioral dimensions, analyzing how cognitive impairment affects dental care utilization across insurance types. It highlights reduced access among those with no insurance or Medicare-only, signaling a need for policy reform. The fourth presentation adopts a life course perspective, exploring how edentulism and social mobility jointly shape cognitive trajectories, with effects varying by age. Collectively, these studies illuminate the bidirectional relationship between oral health and cognitive function, offering actionable insights for prevention, clinical intervention, and policy enhancements to improve older adults’ quality of life.

